# Tobacco advertisement, promotion and sponsorship ban enforcement index at sales points in Panama, 2017

**DOI:** 10.18332/tid/100526

**Published:** 2019-01-29

**Authors:** Víctor Hugo Herrera, Hedley Knewjen Quintana, Cecilio Niño, Beatriz Gómez, Reina Roa

**Affiliations:** 1 Department of Health Technology Research and Evaluation, Gorgas Memorial Institute of Health Studies, Panama City, Panama; 2 Ministry of Health, Panama City, Panama

**Keywords:** tobacco advertisement, promotion, sponsorship, sales points

## Abstract

**INTRODUCTION:**

We assess the tobacco advertisement, promotion and sponsorship (TAPS) ban enforcement in sales points in Panama in 2017.

**METHODS:**

A nationwide observational survey to assess TAPS ban enforcement in sales points was conducted and involved retail sale in non-specialized stores with food, beverages or tobacco predominating according to the International Standard Industrial Classification of All Economic Activities Rev. 4. A TAPS ban enforcement index was developed from factorial analysis by principal component with a polychoric correlation matrix to calculate the mean national index value.

**RESULTS:**

The national TAPS ban enforcement index value was found to be 3.03. The index value in sales points according to the tobacco products advertisement was 1.98, which was significantly lower where the advertisement was present and 3.09 where it was absent (t=7.57, p<0.05). Each of the three health regions corresponding to Indigenous Territories had an index below the national mean: Emberá-Wounáan (2.52), Guna-Yala (2.65), and Ngäbe-Buglé (2.91). Similar findings were observed among health regions with a west national border: Chiriquí (2.80) and Bocas Del Toro (2.93). On the other hand, the top indices were observed in Panama Metro (3.25), Darién (3.53) and Coclé (3.63).

**CONCLUSIONS:**

There is a high level of enforcement of the TAPS ban as a consequence of the full implementation of the FCTC as a law in Panama. However, indigenous territories and west national border areas had the lowest TAPS ban enforcement, making these populations vulnerable. A fertile ground for future research includes the identification of possible vulnerable targets for tobacco products advertisement, particularly in urban areas.

## INTRODUCTION

Panama is one the countries in the Americas with the strictest tobacco control policies^1^. The most important tobacco control policies implemented in Panama include a strong and comprehensive ban on tobacco advertisement, promotion and sponsorship (TAPS), as well as bans on indoor smoking and tobacco product sales to minors, according to the Panamanian Law 13/2008, including supplementary rules^2^. The Selective Tax on the Consumption of Cigarettes and other Tobacco Products was increased from 32.5% to 100% over the retail price in 2009^3^. The funds coming from this tax are used in activities to improve the primary, secondary and tertiary prevention of tobacco use and tobacco-induced diseases, as well as funding customs to hinder illicit trade in tobacco products^3^. Such regulations go beyond the World Health Organization MPOWER package^4^, because the Panamanian government ratified the Illicit Trade Convention^5,6^. The Bill 136, currently under discussion in the Panamanian Parliament will enforce the plain package, ban additives, and increase the number of smoke-free places and licenses for selling tobacco products^7^.

These tobacco control policies have resulted in outstanding economic and public health outcomes^8^. These positive outcomes have been possible due to fiscal and non-fiscal complementary policies. Results from the Panamanian implementation of the Global Adult Tobacco Survey (GATS) show that the prevalence of tobacco product consumption was 6.4% — being the lowest recorded prevalence in the Americas – and 90.4% were aware of the health-related dangers of consumption of these products^9^. However, only 28.5% of the Panamanian population observed TAPS^9^. GATS results regarding TAPS indicate that tobacco industry uses diverse tactics to attract new consumers, e.g. using colorful trademarks and shapes, particularly for young people^10,11^. TAPS are broadcast in both traditional and novel media (social networks, video games, sales points, magazines, search engines, text messages, among others), as well sales points.

The objective of this study was to assess the TAPS ban enforcement at sales points.

## METHODS

A nationwide survey assessing TAPS in sales points was conducted in Panama between the 26 January and 28 April 2016. The calculated sample size was 1532 retail sales points, distributed proportionally using a simple sampling scheme.

**Figure 1:**
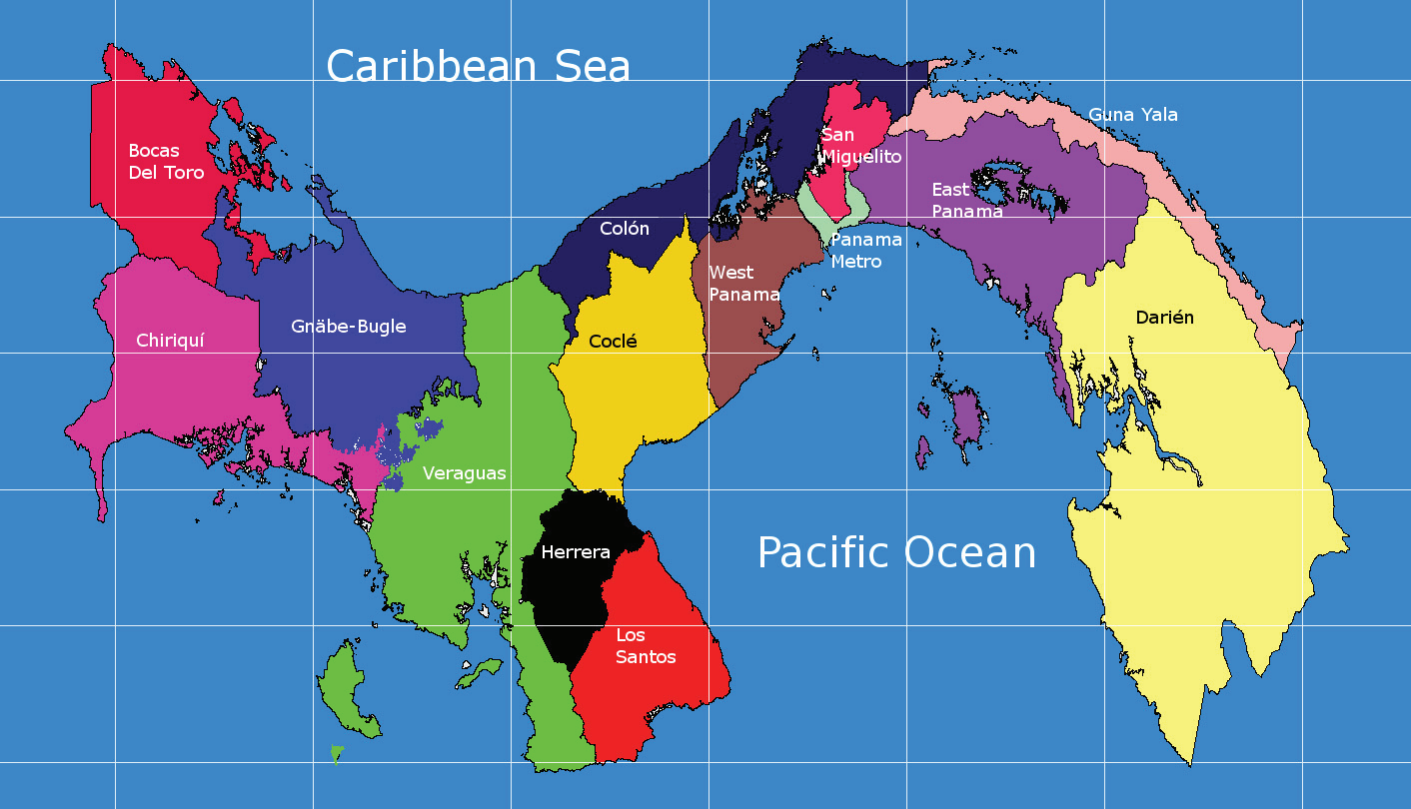
Map of the Health Regions of Panama

Panama is divided into 13 first level administrative divisions, corresponding to 10 provinces: Bocas del Toro, Coclé, Colon, Chiriquí, Darién, Herrera, Los Santos, Panamá, Veraguas and Panamá Oeste (West Panama); and three Indigenous territories: Emberá-Wounan, Ngäbe-Buglé and Guna-Yala. The Health Ministry of Panama divides the country into 16 health regions that correspond to each first level administrative division, except for the province of Panama that is subdivided into four regions: Panama Metro, Panamá Este (East Panama), Panamá Norte (North Panama) and San Miguelito. The Emberá-Wounan Indigenous Territory belongs to the Darien Health Region (Figure 1).

The researchers were public officers working in the respective *Education and Health Promotion* department of the Regional Office of the Ministry of Health where the retail points were located. Each researcher was trained by VHHB and CNH to assess TAPS ban enforcement variables. The researchers were not able to impose a fine at a retail point if the TAPS ban was violated. The researcher required permission of the owner of the retail point to observe the cashier’s section and the surroundings of the retail point entrance. An electronic form developed by CNH was used to record TAPS ban violations.

### Variable definition

The sales-points types were classified according to the *retail sale in non-specialized stores with food, beverages or tobacco predominating* (class 4711 of the International Standard Industrial Classification of All Economic Activities Rev. 4)^12^.

Each retail point was classified with the presence of tobacco products sales if the researcher observed tobacco products (either in the area surrounding the cashier or at his/her request) or a tobacco products display (open or not).

TAPS ban enforcement was assessed with the following variables:

#### Advertisement of tobacco products

This was considered present when at least one of the following was recorded by the researcher:

1. Colors associated with tobacco brands. Such as, Kool brand green color, the sky blue of Viceroy and the red color of Marlboro.
2. Shapes associated with tobacco products brands.
3. Tobacco product packages were publicly visible in an open display.

#### Presence of tobacco consumption

This was recorded by the researcher when there was either tobacco products consumption, presence of any person smoking or cigarette butts in the surrounding entrance area of the retail point.

#### Promotion of tobacco products

This was present if the pollster noticed any gifts, or any kind of discount with the sale of tobacco products.

#### Sponsorship of tobacco products

This was considered present if the pollster recorded posters in the walls of the retail point with any kind of events sponsored by any tobacco product company.

### Statistical analysis

We described the retail points according to their type, Health Region and TAPS ban enforcement variables.

A TAPS ban enforcement index was constructed using a multivariate factorial analysis by principal component with a polychoric correlation matrix. In order to increase the association degree, we used a rotation of the factors using a Varimax method. The Keiser Meyer Olkin statistic (KMO) was calculated to assess the independence between factors. We determined two factors, among them we selected the first, because it had the highest saturation in relation to the variables loadings.

The TAPS ban enforcement index mean value was classified according to retail point type, tobacco sales in the retail point, by Health Region, and nationwide.

Statistical analyses were performed using STATA version 14.0. The cut-off value for a null hypothesis was 0.05.

### Ethics statement

The subjects in the study are retail stores. Therefore, the study does not require ethical permit.

## RESULTS

There were no reports of promotion or sponsorship of tobacco products at retail points.

According to the selling-point type (Table 1), 76.0% of the studied sample belonged to the following three categories: small supermarkets and shops (61.9%), restaurants (8.2%), and supermarkets (5.9%). On the other hand, pharmacies were 5.2% and others 18.8%.

**Table 1:** Type of selling points, according to retail sale in non-specialized stores with food, beverages or tobacco predominating (class 4711 of the International Standard Industrial Classification of All Economic Activities Rev. 4 ). TAPS ban enforcement observational study Panama, 2016

*Type of selling point*	*Number of selling points n (%)*
Small markets and shops	943 (61.9)
Supermarket	90 (5.9)
Restaurants	125 (8.5)
Pharmacist	79 (5.2)
Other*	286 (18.8)

* Other include: gas station, peddler, bars, exclusive retail points, department store, hostel/hotel, bank, barber shop, medical clinic, casino, warehouse store, whole selling, cyber café, laundry, sea food shop and esoteric shop.

Tobacco products were sold in 36.6% of the selling points. Tobacco advertisement was observed in 5.2% of the selling points. The most common tobacco advertisement element was the observation of cigarette packages (4.0%). Nevertheless, some colors related to tobacco trademark products (Kool and Marlboro) were observed in 14.9% of the selling points; the most common color was green, similar to cigarette packages with menthol. In 13.1% of the selling points, a geometric pattern similar to that of cigarette brands was observed. In 2.6% of areas close to the selling point, a person was observed smoking.

As shown in Table 2, tobacco products advertisement was observed in the following types of selling points: small markets and shops (6.7%), supermarkets (7.3%), bars (3.8%), ‘exclusive points of sale’ (14.3%) and whole sales (11%). Advertisement was not observed in other types of selling points.

**Table 2:** Presence of advertisement according to selling points. TAPS ban enforcement observational study Panama, 2016

*Type of selling point*	*Number of selling points with tobacco advertisement n (%)*
Small markets and shops	63 (6.7)
Supermarket	66 (7.3)
Bars	1 (3.8)
Exclusive points of sale	1 (14.3)
Whole selling	1 (11.0)

Tobacco products advertisement was observed in 13.6% of points where tobacco was sold, but was also observed in 0.6% of selling points where it was not sold (p< 0.05).

As shown in Table 3, the proportion of observed persons who smoked was usually higher among selling points where tobacco products advertisement was observed (5.0%) compared to those without an advertisement (2.5%). However, the difference was not statistically significant (χ^2^-test; 1 degree of freedom, p=0.173).

**Table 3:** Presence of tobacco advertisement according to tobacco consumption in sales TAPS ban enforcement observational study Panama, 2016

*Presence of tobacco advertisement*	*Tobacco consumption*		
	*Yes*	*N*	*Total*
Yes	4	76	80
No	36	1407	1443
Total	40	1483	1523

Pearson χ^2^=1.8601 (p-value for 1 degree of freedom=0.173).

Regarding, the factorial model, the Keyser-Meyer-Olkin statistic value was 0.5435. The first factor accumulated 52% of the latent variable. The likelihood-ratio test (independent vs saturated) was statistically significant. The variables that were included in the index are: type of sales point, selling tobacco, tobacco advertisement and tobacco consumption (Table 4).

**Table 4:** Loadings of the variables composing the TAPS index. TAPS ban enforcement observational study Panama, 2016

*Variable*	*Loading*
Selling tobacco	0.9238
Tobacco advertisement	0.8190
Type of selling point	0.6304
Tobacco consumption	0.4247

**Figure 2:**
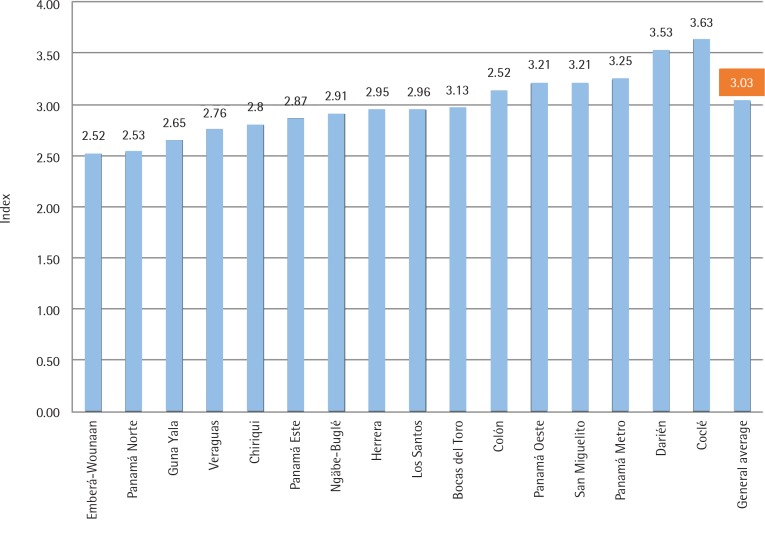
TAPS ban enforcement index of point-of-sales by Health Region, TAPS ban enforcement observational study Panama, 2016

The enforcement of TAPS ban index, stratified according to the presence of tobacco advertisement is shown in Figure 2. The nationwide index was 3.03. The TAPS ban enforcement index mean value was lower in selling points where tobacco advertisement was present (tobacco advertisement present 1.98; tobacco advertisement absent 3.09). The test of averages was compared assuming the same variance with respect to the average index for each category, namely: 1) observed publicity and 2) did not observe advertising. Student’s t-test statistic was 7.57 with p<0.05.

The enforcement of TAPS ban index, stratified according to the health regions is shown in Figure 2. The TAPS ban enforcement index values were below the nationwide value in each of the health regions corresponding to the indigenous territories: Emberá-Wounaán (2.52), Ngäbe-Buglé (2.91) and Guna Yala (2.65); and provinces in the east border Chiriquí (2.80) and y Bocas del Toro (2.96). The top values of the TAPS ban enforcement index were observed in Panamá Metro (3.25), Coclé (3.63), and Darién (3.53).

## DISCUSSION

According to our results, promotion and sponsorship were not recorded for any retail point. Tobacco products advertisement was observed among 5.4% of the retail points. Our results show that there is a high nationwide TAPS ban enforcement in retail points. However, we noticed differences in the TAPS ban enforcement in subnational levels.

Our results showed that Colón, Darien and Coclé had higher TAPS ban enforcement indices than the national index. The results from Darién, might seem unexpected, given that it is mainly rural. However, selling points in Darién are concentrated around more commercial areas within this region, given its poor development.

Other regions, where the index value is higher than the national average had a tendency to have more urban areas making the sales points less disperse, which in turn might have enabled a closer surveillance of the TAPS ban enforcement. Therefore, the geographical features and the local development of retail sales points must be taken into account in order to assess the enforcement of the TAPS ban and is a topic for future research. On the other hand, areas with high density of sales points might represent challenges such as informal retail sales, peddlers and convenience stores in gas stations, where the TAPS ban is difficult to enforce. At such sales points, cigarette packages might be exhibited. In a similar fashion, socioeconomic and ethnic characteristics of the population, in areas where retail sales of tobacco products might take place such as nearby schools and small neighborhoods, are quite complex even if there is high TAPS ban enforcement^13-15^, as in Panama, San Miguelito and Colón. According to a cigarette market survey, about 15.7% of the buyers of tobacco products bought them from peddlers and 12.4% were bought from acquaintances^14^. Several regions in the country have high levels of informal labor that influence inequality regarding the exposure to TAPS, which is higher in highly populated areas with disadvantaged ethnic groups, low economic status and poor development. In such areas, the youth are the main target of TAPS^16-19^. It is important to take into account informal commercial means when assessing the cigarette market, which we could not assess in our study as the International Standard Industrial Classification of All Economic Activities Rev. 412 does not take it into account. However, for difficult to reach areas, such as the indigenous territories, this is not hard to accomplish. Guna Yala Indigenous Territory is a highly dispersed area that can only be totally accessed by sea. The Guna Yala informal workforce reaches up to 84.9% of the population, in Emberá-Wounnan it is about 69.9% and NgäbeBuglé it is about 77.9%^19^. The prevalence of tobacco consumption in indigenous areas reaches 6.9%^9^. There is compelling evidence that tobacco industry focuses its market strategies towards vulnerable groups according to ethnic features, mainly minorities and the youth in selling points close to schools that might increase the risk of early initiation in the latter, particularly underage people^17,20-22^.

Another vulnerable health region where advertisement was mainly observed was Bocas del Toro, which has 54.9% informal employment^19^. It receives a large number of tourists that visit during the whole year and who import their smoking habits to the locals. Bocas del Toro is a semi-urban area with retail sales points set in a smaller and more disperse way than in an urban area. Here, the most common form of advertisement of tobacco products is the open display. The effectiveness of removal of such displays in selling points to try and reduce the prevalence of smoking is not clear and warrants further research^23,24^.

Despite the high level of enforcement of the TAPS ban, our results reveal that some social determinants and factors, such as TAPS exposure among vulnerable populations, are serious threats to effective tobacco control needed to maintain the outstanding low prevalence in Panama. Better means to control TAPS, which is the cornerstone of tobacco products marketing^10,18^, are needed.

The enforcement of TAPS ban requires continuous surveillance by the health authority in Health Regions with the lowest TAPS ban index.

Our findings show that tobacco packages that are publicly visible are the main component of tobacco products advertisement because they are placed in semi-opened displays. In addition, colors and shapes associated with tobacco products brands were also observed.

An important strength of our study is that the variables used in the index were sufficient to estimate and establish a comparative measure regarding the TAPS ban enforcement at a national and subnational level. The factorial analysis has been shown to be a trustworthy method to assess different types of variables in order to synthesize a unique index to evaluate TAPS compared to other descriptive methods. Such a method has been used in other contexts to synthesize several variables into a simple parameter^25-27^.

However, a limitation of this method is that it is not possible to include variables without adequate information that is needed to obtain a more robust index.

## CONCLUSIONS

There is a high level of TAPS ban enforcement in sales points in Panama. In 5.2% of the sales points, there was advertisement of tobacco products. It is noteworthy that these advertisement items included the exposure of cigarette packs in opened displays, the presence of colors and geometric patterns suggesting tobacco products trademarks, such as Kool menthol cigarettes and Marlboro Red. Our results indicate that indigenous territories have low enforcement of the TAPS ban because they are low population density areas. However, in urban areas with high level of TAPS ban enforcement, there is a complex situation regarding ethnicity, poverty, and informal labor, which might translate into an inadequate ban on advertisement exposure in sales points targeting vulnerable populations, particularly the youth attending schools near sale points. It is necessary to pay attention to these elements that might become the biggest threat to achieving tobacco control policies in the long-term.
